# A systematic review of missed opportunities for improving tuberculosis and HIV/AIDS control in Sub-saharan Africa: what is still missed by health experts?

**DOI:** 10.11604/pamj.2014.18.320.4066

**Published:** 2014-08-21

**Authors:** Basile Keugoung, Florent Ymele Fouelifack, Richard Fotsing, Jean Macq, Jean Meli, Bart Criel

**Affiliations:** 1 Ministry of Public Health, Cameroon; 2 Research, Education, and Health Development Group (GARES-Falaise), Dschang, Cameroun; 3 Yaoundé Central Hospital, Yaoundé, Cameroon; 4 Institut de Recherche Santé et Société, Université Catholique de Louvain, Brussels, Belgium; 5 Faculty of Medicine and Biomedical Sciences, University of Yaoundé I, Yaoundé, Cameroon; 6 Public Health Department, Institute of Tropical Medicine, Nationalstraat Antwerp, Belgium

**Keywords:** Missed opportunities, HIV/AIDS, tuberculosis, sub-Saharan Africa, health systems

## Abstract

In sub-Saharan Africa, HIV/AIDS and tuberculosis are major public health problems. In 2010, 64% of the 34 million of people infected with HIV were reported to be living in sub-Saharan Africa. Only 41% of eligible HIV-positive people had access to antiretroviral therapy (ART). Regarding tuberculosis, in 2010, the region had 12% of the world's population but reported 26% of the 8.8 million incident cases and 254000 tuberculosis-related deaths. This paper aims to review missed opportunities for improving HIV/AIDS and tuberculosis prevention and care. We conducted a systematic review in PubMed using the terms ‘missed’(Title) AND ‘opportunities’(Title). We included systematic review and original research articles done in sub-Saharan Africa on missed opportunities in HIV/AIDS and/or tuberculosis care. Missed opportunities for improving HIV/AIDS and/or tuberculosis care can be classified into five categories: i) patient and community; ii) health professional; iii) health facility; iv) local health system; and v) vertical programme (HIV/AIDS and/or tuberculosis control programmes). None of the reviewed studies identified any missed opportunities related to health system strengthening. Opportunities that are missed hamper tuberculosis and/or HIV/AIDS care in sub-Saharan Africa where health systems remain weak. What is still missing in the analysis of health experts is the acknowledgement that opportunities that are missed to strengthen health systems also undermine tuberculosis and HIV/AIDS prevention and care. Studying why these opportunities are missed will help to understand the rationales behind the missed opportunities, and customize adequate strategies to seize them and for effective diseases control.

## Introduction

Sub-Saharan Africa is the world's region most affected by HIV/AIDS and tuberculosis (TB). In 2010, 64% of the 34 million of people infected by HIV were reported to be living in sub-Saharan Africa [1]. Despite investments and efforts done so far, only 41% of eligible HIV-positive people had access to antiretroviral therapy (ART); only 35% of pregnant women underwent testing for HIV, and only 48% of these HIV-positive pregnant women had access to effective antiretroviral therapy to prevent mother-to-child transmission. Additionally, only 23% of HIV-positive children had access to ART. This low access to prevention and treatment contributed to 2.7 million new HIV infections in 2010. Regarding TB, in 2010, sub-Saharan Africa had 12% of the world population but reported 26% of the 8.8 million incident cases -the world's highest incidence at 256 cases per 100000 inhabitants- and 254000 TB-related deaths. Sub-Saharan Africa counted for 82% of HIV-positive people with TB, and is the sole WHO world region that is not on track to halve the 1990 TB mortality by 2015 [2].

In recent years, there has been a significant increase in global health investment dedicated to disease control programmes in low-income countries. Indeed, official development assistance for health for the Organization for Economic Cooperation and Development increased from an average of 2.8 billion USD per year in 1980-1990 to almost 6.4 billion USD per year in 2002-2006 -reaching 13.3 billion USD in 2006. HIV/AIDS control activities account for 32% of total funding, and HIV/AIDS, malaria and other communicable diseases, such as TB, account for nearly half (47%) of total official development assistance for health in 2002-2006 [3]. More results could be achieved with regards to these resources. Unfortunately, developed countries supporting these activities currently face financial crises that may undermine the continuity and sustainability of funding. This even led the Global Fund to cancel its 11th round for the fight against HIV/AIDS, TB and malaria [4]. Furthermore, besides infectious diseases, new threats that also require urgent attention, such as non-communicable chronic diseases and global warming, are emerging while health resources are limited and scarce and health systems remain weak.

In high income countries, the quality of the health systems contributed to offer universal access to health care to people living with HIV/AIDS and/or suffering from TB, and to significantly reverse the epidemic. On the contrary, in sub-Saharan Africa, the extended and persistent HIV/AIDS pandemic and the recrudescence of TB reveal huge uncovered needs of the population by health systems. Some studies investigated constraints faced by African health systems in delivering effective interventions for the control of major infectious diseases, and have proposed strategies to tackle those constraints [5, 6]. Consequently, the persistent high morbidity and mortality related to HIV/AIDS and TB could be the result of opportunities not seized to timely deliver adequate and effective care against these conditions [7].

The objective of this study was to identify, through a systematic review of the literature, missed opportunities (MOs) to deliver effective care to prevent, diagnose and treat HIV/AIDS and TB in sub-Saharan Africa. Knowing these MOs could help health systems stakeholders to develop strategies for actually reversing the two deadly epidemics.

## Methods

We searched PubMed on September 9, 2012, using the terms ‘missed’ (Title) AND ‘opportunities’ (Title). We found 490 articles which were then independently reviewed by two investigators. The analysis started with the title, then by the abstract and finally by the full content. When there was no consensus, the full text of the article was analyzed. The study received ethical clearance from the National Ethics Committee of Cameroon (N° 258/CNE/SE/2011) and the Institutional Review Board of the Institute of Tropical Medicine, Antwerp, Belgium (Ref 11 21 5 773).

### Study selection

**Figure 1:**
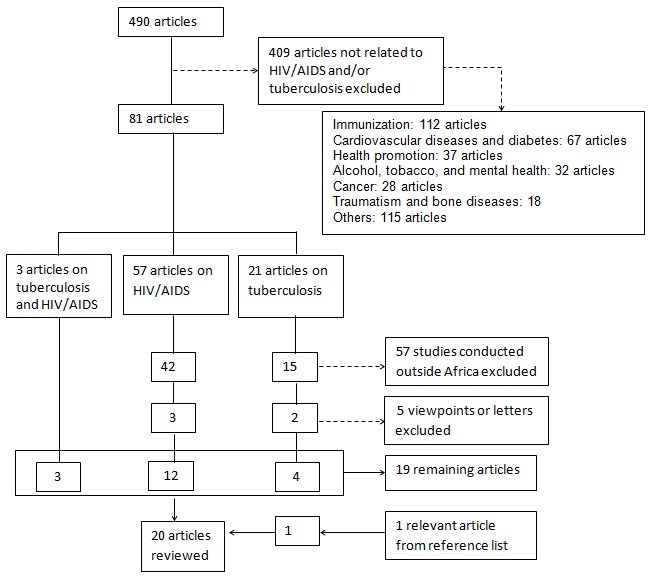
Search strategy

We included systematic review and original research articles done in sub-Saharan Africa on MOs in HIV/AIDS and/or TB care. Viewpoints, letters, and studies conducted outside sub-Saharan Africa were excluded. Date of publication and language were not considered as criteria for exclusion. Initially, 409 articles with topics not related to HIV/AIDS and TB were excluded through the review of their titles (Figure 1). Then the abstracts of the remaining 81 articles were analyzed: 57 on HIV/AIDS, 21 on TB and 3 on TB and HIV/AIDS.

Of the 57 articles related to HIV/AIDS, 42 studies conducted outside Africa and 3 viewpoints were excluded while 12 were relevant for the review. Of the 21 articles related to TB, 15 studies conducted outside Africa and 2 viewpoints were excluded, 4 articles were relevant and one additional article was identified from the reference list. All three studies on HIV/AIDS and TB were relevant. A total of 20 articles were therefore reviewed.

### Data extraction

Data were extracted independently by two investigators using a standardized extraction sheet. Data extracted included the characteristics of the study (setting, type, objective, data collection period, publication date) and the MO. Missed opportunities were further inductively classified into five categories: patient and community; health professional; health facility; local health system; and vertical programme (HIV/AIDS and/or TB control programmes).

### Current status of knowledge

The 20 relevant articles included in the review were 18 original articles and 2 systematic reviews. The studies were conducted in 6 English-speaking southern and eastern sub-Saharan African countries with high HIV prevalence: Ethiopia, Kenya, South Africa, Tanzania, Uganda and Zambia. The articles were published between 1993 and 2012. Regarding HIV/AIDS, opportunities were not seized to detect HIV (among children from HIV-positive mothers, pregnant women, and general population), to identify people eligible for antiretroviral therapy (ART), or to offer ART for the prevention of mother-to-child transmission of HIV or to eligible HIV-positive persons. For TB, opportunities were not taken to detect TB in suspect patients, to offer chemoprophylaxis to exposed children, to diagnose TB in symptomatic patients, or to create in health facilities conditions for optimal detection of TB.

### Missed opportunities in HIV/AIDS prevention and care, Table 1

*Patient, local organization and community level:* At the level of the patient, MO for improving HIV/AIDS prevention and care (Table 1) were related to consultation delays [8, 9]; behavioural risks exposing people to HIV infection [8, 10]; consultation in structures without equipment for HIV screening (e.g. drug shops, traditional healers) [8, 11, 12]; low perception of HIV infection risks; refusal of HIV testing [8, 11, 13–15]; low adherence to ART; and loss to follow up [9, 16, 17]. At the community level, MOs were associated with the lack of community and psychosocial support groups [12]; negative or neutral messages of religious organizations such as cure of AIDS through faith [18]; stigmatization of people living with HIV/AIDS [18]; and low membership of people living with HIV/AIDS to community support organizations [18].

**Table 1:** Articles reviewed and summary of causes of missed opportunities for HIV/AIDS prevention and care

Authors	Country	Study type	Year of publication	Data collection period	Causes of missed opportunities
Myer et al[10]	South Africa	Cross-sectional	2007	August-November 2005	1) low perception of HIV risks by some patients; 2) risk behaviors; 3) non-integration of other health care activities in ART programmes
Rispel et al[12]	South Africa	Cross-sectional	2009	April - July 2007	1) workload 2) No support group for HIV positive women; 3) no awareness of mother-to-child transmission of HIV directives; 4) no guidelines for the prevention of mother-to-child transmission of HIV in health facilities; 5) stock-out of HIV drugs, reagents and other medical supplies; 6) staff shortages; 8) demotivation of staff; 9) lack or poor quality training and supervision of health staff and community health workers; 10) no HIV testing for some patients; 11) non-referral of HIV-positive patients to adequate health services for care
Kharsany et al[13]	South Africa	Cross-sectional	2010	July 2005 –June 2006	1) no HIV testing for some patients; 2) refusal of HIV testing by patients; 3) counsellors not available
Louwagie et al[15]	South Africa	Retrospective cohort study	2012	October 2008 – March 2009	1) lack of HIV/AIDS care unit in the health facilities; 2) no HIV testing for some patients
Perumal et al[20]	South Africa	Policy analysis	2009	2009	1) no HIV testing for some patients; 2) low competence of health staff on HIV/AIDS care; 3) no prescription of ART to eligible persons; 4) Little implementation of preventive measures in the opt-out HIV testing approach; 5) parallel and disconnected HIV/AIDS and tuberculosis services; 6) little implementation of measures to improve ART adherence
Nkonki et al [9]	South Africa	Cross-sectional	2007	April-June 2005	1) no attendance to antenatal care; 2) no HIV testing for some pregnant women; 3) HIV testing result not delivered to patients; 4) ART was not prescribed to HIV-positive pregnant women; 5) Incorrect instructions for antiretroviral drugs intake
Wettstein et al[16]	Sub-Saharan Africa	Systematic review	2012	January 2002 – March 2012	1) no HIV testing for some pregnant women and some children born from HIV-positive mothers; 2) prescription of less effective antiretroviral schemes for the prevention of mother-to-child transmission of HIV; 3) loss to follow-up; non-initiation of ART for eligible HIV-positive pregnant women and infants born from HIV-positive mothers
Park-Wyllie et al[22]	Sub-Saharan Africa	Systematic review	2002	1996-1999	1) toxicity and intolerance of available antiretroviral drugs; 2) HIV resistance to available antiretroviral drugs
Fetene et al[14]	Ethiopia	Cross-sectional	2010	November -December 2008	1) no HIV testing for some patients; 2) refusal of HIV testing by patients; 3) reduced perceived risks
Njeru et al [21]	Kenya, Tanzania, Zambia	Cross-sectional	2011	2007-2008	1) lack of counselling especially for HIV negative persons in opt-out HIV testing model; 2) limited preventive measures for people with HIV negative test
Watt et al [18]	Tanzania	Cross-sectional	2009	October 2006 – February 2007	1) negative or neutral messages delivered by church organizations; 2) stigmatization of people living with HIV/AIDS
Tribble et al [19]	Tanzania	Cross-sectional	2009	November 2003 - January 2006	1) no HIV testing for some tuberculosis patients
Watson-Jones et al [17]	Tanzania	Cross-sectional	2012		1) low adherence to ART; 2) non-initiation of ART for eligible HIV-positive persons; 3) non-referral of HIV-positive persons to HIV clinics
Wanyenze et al[8]	Uganda	Cross-sectional	2011	May 2008-Mars 2010	1) care in non-medical settings; 2) no HIV testing for some patients; 3) risks behaviour; 4) consultation delays
Larsson et al[11]	Uganda	Prospective cohort study	2012	Mai 2008-March 2010	1) Failure to diagnose HIV infection for infected persons who attended medical clinics; 2) consultation in settings without equipment for HIV testing (pharmacy, drugs shops); 3) expansion of provider-initiated HIV testing to all health units; 4) parallel and disconnected units for HIV testing and care; 5) HIV testing only in health facilities (those who are not ill will not be tested); 6) Targeting of mainly public facilities (29% of health care in Uganda) by ART programmes; 7) low perception of being infected by HIV

*Health professionals:* Regarding health professionals, most studies noted that HIV testing was not proposed to patients [8, 9, 11–16, 19, 20]. Some MOs were due to the lack of skills on HIV/AIDS care [9, 12]; the ART not proposed to eligible people [9, 12, 15–17, 20]; the non-referral of HIV-positive persons to HIV clinics for care [17]; the lack of counselling and implementation of preventive measures, especially in opt-out testing strategy [20, 21]; the lack of permanence of service for HIV counseling and testing [12, 13]; the lack or poor quality of training and supervision leading to staff demotivation [12]; and the low implementation of measures for improving compliance to treatment [17, 20].

*Health facilities:* In health facilities, MOs were secondary to stock-outs of tests, drugs and other medical supplies undermining the continuity of care [12]; unavailability of directives on HIV/AIDS care; the scarcity of qualified staff leading to high workload; and the lack of adequate equipment [12]. Another cause for MO was the non-integration of other health care activities in HIV/AIDS programmes [10].

*Local health systems:* In local health systems, MOs were due to the fragmentation of HIV/AIDS care in multiple units (HIV screening, ART, prevention of mother-to-child transmission of HIV); the lack of HIV/AIDS care units in some health facilities [15]; and the existence of parallel and disconnected systems of HIV/AIDS and TB care [20].

*HIV/AIDS programmes:* Concerning HIV/AIDS programmes, MOs were related to the irregular supply of HIV tests, pre-therapeutic reagents, and drugs; the inadequate equipment provided to health facilities; and the poor maintenance of the equipment [11]. Additionally, other MOs were secondary to the targeting of only a few health facilities, especially public facilities to deliver HIV/AIDS care [11]; the directives of HIV/AIDS care centered on (1) confidentiality and limiting community support interventions [10], and (2) promoting opt-out strategies for HIV testing without counseling pre- and post-test and implementation of preventive measures, especially for HIV-negative persons [21]; and the resistance of HIV to and toxicity of available antiretroviral drugs [22].

### Missed opportunities in TB prevention and care, Table 2

*Patient, local organization and community level:* Like for HIV/AIDS, patient-related MOs for improving TB control (Table 2) were secondary to the long-waiting delays before seeking care [23] and consultation in settings without equipment for TB diagnosis, such as drug shops and traditional healers [23].

**Table 2:** Articles reviewed and summary of causes of missed opportunities for tuberculosis prevention and care

Authors	Country	Study type	Year of publication	Data collection period	Causes of missed opportunities
Perumal et al [20]	South Africa	Policy analysis	2009	2009	1) Little adaptation of programme strategies in relation with tuberculosis epidemiological changes; 2) no investigation of tuberculosis in high risk patients; 3) parallel and disconnected HIV/AIDS and tuberculosis services; 4) little implementation of measures to improve compliance to tuberculosis treatment
Louwagie et al [15]	South Africa	Retrospective cohort study	2012	October 2008 – March 2009	1) lack of tuberculosis care in some health facilities; 2) parallel and disconnected HIV/AIDS and tuberculosis services
Tribble et al [19]	Tanzania	Cross-sectional	2009		1) failure to detect tuberculosis in HIV positive person with symptoms of active tuberculosis
Creswell et al [24]		Systematic review	2011	…-May 2010	1) Neglect of risk factors of tuberculosis infection (alcohol, tobacco, chronic non-communicable diseases); 2) no investigation of tuberculosis in high risk patients
Du Preez et al[25]	South Africa	Cross-sectional	2011	March 2003 - February 2007	1) no chemoprophylaxis against tuberculosis for children exposed to a patient with active pulmonary tuberculosis and to HIV-positive children; 2) no investigation of tuberculosis in HIV-positive and exposed children
Gie et al [26]	South Africa	Cross-sectional	1993	September-December 1990	1) no investigation of tuberculosis in exposed children; 2) missed tuberculosis diagnosis in children with signs and symptoms of active tuberculosis; 3) no investigation of tuberculosis in exposed and suspect children
Field et al [27]	South Africa	Cross-sectional	2011	2003-2007	1) missed tuberculosis diagnosis in patient with signs and symptoms of active tuberculosis; 2) little investigation of signs and symptoms of tuberculosis among patients; 3) poor quality of care (lung, weight and lymph nodes were not examined); 4) paraclinical exams were not carried out (sputum smear and culture, chest radiography, lymph node aspiration…)
Sendagire et al [23]	Uganda	Cross-sectional	2010	April 2007 -April 2008	1) failure to investigate tuberculosis by health professionals; 2) consultation in settings without equipment for tuberculosis diagnosis (drugs shop, traditional healers); 3) consultation delays; 4) lack of tuberculosis services in some health facilities (especially private)

*Health professionals:* Regarding health professionals, the MOs for TB care were associated with the inattention to TB risk factors, such as tobacco, alcohol, and some chronic diseases (diabetes) [24]; the lack of investigation of TB in high-risk patients [19, 20, 23–27]; and the missed diagnosis of TB in patients with signs and symptoms of TB [26, 27]. Other MOs were linked to the lack of laboratory examinations, such as sputum culture, gastric tubage in children, and chest radiography, to facilitate TB diagnosis [26, 27]. The MOs related to treatment were due to the limited implementation of preventive measures for improving treatment adherence [20], and the absence of chemoprophylaxis to under-five children exposed to a patient with smear-positive pulmonary TB [25, 26].

*Health facilities:* The main MO was secondary to the lack of a TB care unit in some health facilities, increasing missed diagnoses if suspect patients are not referred to adequate facilities [15, 23].

*Local health system:* The low ART coverage in high-HIV-prevalence countries [20] and the existence of independent and disconnected systems for TB and HIV/AIDS programmes leading to a wastage of scarce health resources and an increased burden on patients resources [15, 20] were identified as causes of MO at this level.

*TB control programme:* It was noted that very minimal adaptation of TB control programme strategies in relation to TB epidemiologic changes (such as increased TB cases in women and multidrug-resistant TB) was done [20].

### Seizing opportunities

This review identifies MOs to improve prevention, detection, diagnosis and adequate care of HIV/AIDS and TB in sub-Saharan African health systems. The limit of this study is that articles were searched only on PubMed while other literature, such as projects reports and programme evaluations, could also provide useful information on MOs. Additionally, studies reviewed were mainly conducted at health facility and community levels whereas other MOs for improving TB and HIV/AIDS control could be related to higher levels of the health system, to health policies, to international procedures of international organizations funding these programmes, and to the quality of the interface between HIV/AIDS and TB control programmes and general health services. However, this study identified sizeable factors that could explain why TB and HIV/AIDS have not yet been controlled in sub-Saharan Africa in spite of the availability of effective interventions.

MOs that are described in sub-Saharan Africa do exist in other settings, but at a much lower scale, indicating that these opportunities can be seized to improve TB and HIV/AIDS care. In developed countries, the low proportion of MOs leads to a much lower incidence and to a high detection rate of HIV and TB cases. For example, a study found that between 1996 and 2000, among 4287 HIV-positive pregnant women who delivered in six hospitals in the United States, 92% followed antenatal care and did an HIV testing before delivery and the caesarian section rate for the prevention of mother-to-child transmission of HIV increased from 20% in 1996 to 48% in 2000. Additionally, in 2000, 87% of HIV-positive pregnant women received effective ART schemes for the prevention of mother-to-child transmission of HIV [28]. On the contrary, even though significant progress have been achieved, only 35% of pregnant women did their HIV testing and only 48% of HIV-positive pregnant women received the effective ART scheme for the prevention of mother-to-child transmission of HIV in sub-Saharan Africa in 2010 [1]. Moreover, in Kenya, caesarian section as an indication of the prevention of mother-to-child transmission of HIV was performed only in 14% of HIV-positive pregnant women in 2010 at two National hospitals recognized as the leading model institutions for the prevention of mother-to-child transmission of HIV [29]. In Cameroon, the ART coverage among HIV-positive pregnant women was only 20% in 2011 [30].

Therefore, the persistence of TB and HIV/AIDS epidemics in sub-Saharan Africa could be the result of MOs. By seizing these opportunities, the prevention, early detection and care for these conditions could be improved. Each MO reduces access to effective TB and HIV/AIDS care [31]. These MOs concern all levels of the health system, namely populations and patients, health professionals, health facilities, local health systems, and TB and HIV/AIDS control programmes. Consequently, controlling TB and HIV/AIDS fundamentally requires seizing opportunities and/or avoiding that these opportunities are missed. Therefore, strategies to improve the control of TB and HIV/AIDS should involve populations, patients, and local organizations at the community level; health professionals delivering care at the facility level; managers of general health services and of vertical programmes; and lastly policymakers. Also, donor partners and international organizations funding and supporting vertical programmes are important stakeholders that are needed for implementing these opportunities.

Additionally, some MOs for improving HIV/AIDS and TB care have consequences that go beyond TB and HIV/AIDS diseases. By not developing integrated care (for example TB and HIV/AIDS collaborative care) complexifies patient itineraries and increases barriers to health care [20], and leads to fragmentation and inefficient use of scarce health resources [32]. As such, the lack of reproductive care in HIV/ADS services limits access to family planning for people living with HIV/AIDS [10]. This may increase unplanned pregnancies and neonatal and maternal morbidity and mortality in settings such as sub-Saharan Africa, where there is no universal access to health care. Other MOs are common to TB and HIV/AIDS care [19]. The opportunities missed secondary to the lack of equipment and the scarcity of skilled staff in health facilities impair both TB and HIV/AIDS care, including those related to other vertical programmes. Missed opportunities are not unique to TB and HIV/AIDS, and many studies also described MOs for adequate care for other health problems in sub-Saharan Africa, such as immunization [33, 34], family planning and reproductive health [35] and syphilis treatment during antenatal care [36]. Globally, MOs reflect constraints faced by sub-Saharan African countries to deliver quality health care [37]. These constraints affect all health system components [38, 39]. If general health systems do not function, there is a high risk that disease control programmes will also fail to deliver [40].

Therefore, seizing opportunities goes far beyond TB and HIV/AIDS control programmes and encompasses measures to improve the quality of care and the performance of the whole health system. Indeed, strong health systems are a prerequisite for achieving disease control programme objectives as well as health goals [6, 41–43]. The current weak health systems in sub-Saharan Africa contribute to a vicious circle of ‘weak health systems → poor health indicators → overburdened health services → weak health systems’. Consequently, some MOs would not exist or would be significantly minimized if health systems were strengthened. In strong health systems, resources are adequately used to respond to population needs. Therefore, what are missing in the reviewed studies are MOs that were not seized to actually strengthen health systems in sub-Saharan Africa.

## Conclusion

Many opportunities for improving TB and HIV/AIDS prevention and care are not seized by patients, populations, communities, health professionals, and managers of general health services and of vertical programmes. Missing such opportunities reduces prevention, early detection and treatment of TB and HIV/AIDS, and thus undermines the control of both diseases in sub-Saharan Africa. However, what is still missing in the analysis of health experts is the identification of health system strengthening's missed opportunities as a leading factor undermining disease control in sub-Saharan Africa. Studying why these opportunities are missed will help to understand the rationales behind the MOs, and customize effective strategies for seizing them.
